# Quality of life and health-related utility after trans-oral surgery for head and neck cancers

**DOI:** 10.1186/s12955-021-01836-3

**Published:** 2021-11-03

**Authors:** Enea Parimbelli, Christian Simon, Federico Soldati, Lorry Duchoud, Gian Luca Armas, John R. de Almeida, Silvana Quaglini

**Affiliations:** 1 Department of Electrical, Computer and Biomedical Engineering, University of Pavia, Pavia, Italy; 2 Department of Otolaryngology - Head and Neck Surgery, CHUV, UNIL, Lausanne, Switzerland; 3 Department of Otolaryngology, Hospital L. Mandic Merate, ASST Lecco, Merate, Italy; 4 Princess Margaret Cancer Centre, University Health Network, Toronto, Canada; 5 Department of Otolaryngology-Head and Neck Surgery, University of Toronto, Toronto, Canada

**Keywords:** Cost-utility analysis, Utility elicitation, Head & neck cancer, Standard gamble, Rating scale

## Abstract

**Purpose:**

The purpose of this study was to assess utility coefficients of health states following two minimally invasive surgical approaches for head and neck cancer, namely trans-oral robotic surgery and trans-oral laser microsurgery. Those utility coefficients will be later exploited in an economic evaluation study comparing the two approaches.

**Methods:**

The above cited economic evaluation will be done from the Swiss healthcare system perspective and, as such, Swiss healthcare professionals were interviewed to elicit utility coefficients. Health states, ranging from remission to palliative care, were described using clinical vignettes. A computerized tool (UceWeb) implementing standard gamble and rating scale methods was used.

**Results:**

Utility coefficients for 18 different health states were elicited with the two methods from 47 individuals, for a total of 1692 values. Elicited values varied from 0.980 to 0.213. Comparison with values elicited in previous studies show the need for population-specific elicitation, mainly for the worst health states.

**Conclusion:**

Herein we report health utility coefficients for the Swiss population for health states following minimally invasive trans-oral surgery. This study provides utility values that can be used not only for a specific cost-utility analysis, but also for future studies involving the same health states.

**Supplementary Information:**

The online version contains supplementary material available at 10.1186/s12955-021-01836-3.

## Background

In head and neck surgery minimally-invasive techniques have recently been introduced to reduce access-related morbidity and their associated functional deficits. Such novel approaches are mostly used to access cancers in the oropharynx [1, 2]. Trans-oral approaches such as trans-oral laser microsurgery (TLM) and trans-oral robotic surgery (TORS) have provided improved visualization for tumors previously amenable only to transmandibular or other ‘open’ surgical approaches [3]. While the oncologic principles for treating head and neck cancers remain the same regardless of the approach, the costs related to various approaches are different. As such, an economic evaluation is needed to inform and guide decision making about adoption of techniques.

Our group has developed a model for comparing those two surgical approaches through an economic evaluation, and precisely a cost-utility analysis (CUA) [4–6]. The model has been developed as a decision tree [7] embedding Markov processes [8] to represent a patient’s transitions among different health states following trans-oral surgery [9]. Direct costs associated with every intervention and every health state treatment have been included in the model, while quality-adjusted life years (QALYs) are used as primary health outcome [10]. To calculate QALYs, a patient’s expected survival is split in time intervals, each one (Ti) spent in a specific condition Ci. Assuming that Ci is associated with a utility coefficient (UC) UCi, QALYs are computed as the weighted sum of Ti × UCi. UCs range from 0 to 1, where 0 is for the worst possible health state (usually death) and 1 is for the best possible one (perfect health). Thus, a UC quantifies a patient’s preference for a given health state.

Different methods have been proposed in the literature for eliciting UCs for sub-optimal healh states. A complete review of the utility elicitation methods is beyond the scope of this paper and can be found in [11]. In the following we introduce the most popular ones, namely the standard gamble (SG) method, the Time Trade-Off (TTO) and the rating scale (RS). In the SG, based on the axioms of utility theory [12], the interviewee must envision a hypothetical scenario in which he must choose between living the remainder of his life in the sub-optimal *S* state or accepting a gamble in which the outcome may be perfect health or alternatively sudden death. The gamble may be presented as a hypothetical treatment (e.g., a surgical intervention or a drug) that may result in perfect health, but presents a risk *p* of death, due to complications or toxicity for example. The first risk value proposed can be arbitrary (e.g., 0.5), and then varied (increased or decreased according to the answers) until the interviewee can’t decide about the gamble. At that point, the UC is computed as (1–*p*). In the TTO method, the patient is asked to choose between living his entire remaining life (*t*1) in the state S or to live shorter (*t*2 < *t*1) but in a perfect health state. Like the probability *p* in SG, the amount of time a patient is proposed to give up in order to heal completely (i.e., *t1-t2*) is varied until the patient is indifferent between the two choices. The UC is then calculated as *t*2/*t*1. The RS method, conversely, is simply calculated by asking a respondent to rate S on a scale between 0 and 100, then normalizing to the range 0–1. Values derived from RS are not true UCs as they fail to meet the utility theory requirement of “decision under uncertainty” [13]. However, they may help to validate utilities derived by other methodologies and are often easier to comprehend. Finally, also indirect utility elicitation methods, based on questionnaires, are widely used. The most popular example is through the administration of the EQ-5D questionnaire and its conversion in a UC (note that this conversion may lead to values less than zero, i.e., states considered worse than death).

For what concerns utility elicitation in head and neck cancer, a systematic literature review was previously published [14]. Meregaglia et al. state that “there is currently a lack of research for some disease phases including recurrent and metastatic cancer, and treatment-related complications”, which is specifically what we target in our article, i.e., how quality of life is affected by early and late consequences of trans-oral surgery. Indeed, only one study by de Almeida et al. [15] reported UCs related to a number of health states following TORS or radiotherapy in oropharyngeal cancer patients. In that study, UCs were elicited using the SG and RS methods. UCs for 21 health states, presented through scenarios, were elicited from 50 healthy subjects belonging to the North-American population. UCs for head and neck cancer have been elicited also by Noel et al. [16] in Canadian population. However, their aim was to investigate face and construct validity of different elicitation methods, and no specific post-surgery states were addressed. Finally, Liao et al. [17] compared utility values ascertained from Taiwan and Sweden, and suggested the need for understanding population differences, which is corroborated by Caulley et al. [18]. As a matter of fact, UCs are subjective measures, and may vary among different populations, depending on cultural, geographical, and economical factors [19, 20]. Thus, in principle, UCs should be elicited from the same population for which the economic analysis is intended. Unfortunately, utility elicitation is a very time consuming and challenging task, and often researchers rely on values found in the literature, even if these values have been collected from different populations. To avoid this methodological pitfall in our economic analysis, we aimed to ascertain UCs directly from members of the Swiss population. Notably, no study in the literature targeted Swiss population, and no study other than [8] reported UCs for the health states after minimally invasive surgery.

Thus, this paper presents the methodology adopted for eliciting UCs, and the actual UCs associated to the health states represented in the above mentioned decision tree for CUA.

## Methods

### Study population

We performed a cross-sectional study where we interviewed a set of 47 healthcare professionals on a voluntary basis from the Otolaryngology division of Centre Hospitalier Universitaire Vaudois (CHUV) located in Lausanne, Switzerland. We prepared a series of factsheets describing all the health states considered in the study (see also additional material). The preparation of the scenario sheets involved feedback-refinement iterations among the clinical experts from CHUV. The final version of the sheets has been used during the interviews with all the volunteers.

To calculate the interviewees’ sample size, we considered estimating the mean value of UCs with a precision (margin of error) of 0.05, assuming a standard deviation of 0.15. To achieve a confidence level of 95%, the minimum number of persons needed is 38 [21]. To be conservative, and to prevent loss of statistical power due to drop-out or data issues, we enrolled 47 volunteers.

### The computerized tool for utility elicitation

Utility elicitation is a challenging task. In order to obtain consistent values from all the individuals, it is important that everybody is interviewed following the same procedure. We used a computer based tool, UceWeb, a system developed by our group [4, 22, 23] and that has been previously validated in other medical contexts [24, 25]. The main features of UceWeb are:

(1) a graphical interface implementing several methods for utility elicitation;

(2) a decision support facility suggesting the best elicitation method according to the interviewee’s and health state characteristics;

(3) a common terminology for the health states (SNOMED) for which UCs are elicited (additional states, not covered by SNOMED, may be added if necessary);

(4) a collaborative environment where different researchers can feed the UCs repository, while providing a basic profile for every interviewed individual (age, gender, country, if he is a patient or not, and other features known to affect an individual’s preferences). This will allow future studies to have larger and larger sets of UCs for specific target populations. The study described in this paper contributed to this repository indeed;

(5) a direct link to TreeAge Pro[26], allowing to run a decision tree just after having elicited UCs from a single patient or having retrieved a UC set for a target population from the repository;

(6) UceWeb is a multiuser (and multilingual) system and this allowed the interviewers to work in parallel and save time. For the current study, French language has been used.

### Utility elicitation sessions

The elicitations were done in 9 sessions over a 101 days period. As mentioned, all the interviews were done using UceWeb and supported by the same set of factsheets describing the clinical context, the treatments and their possible consequences. In this way, all the volunteers received the same information in the same format. Two physicians (FS and LD) performed all the interviews and answered all the volunteers’ questions. First of all, the clinical problem was described, relying on available literature [27–29]. In the following textbox we report the exact words used for informing the volunteers at the beginning of each interview, which is useful for the reader as well, to understand the context, while omitting some details for sake of space.

After describing the clinical problem, the interview is introduced through a hypothetical *shared decision-making framework:*

Imagine being a patient who consults an otolaryngologist following the diagnosis of cancer of the oropharynx. Your doctor will describe different treatment options and you will finally choose the option that best suits your wishes. After sufficient discussion with him, you will have enough information about the risks and benefits to be able to make a decision.

After this introduction, the elicitation exercise begins. First of all, the oncologist describes the scenario #1, depicting the first month consequences of the surgical intervention, which are the same for TORS and TLM, and then he describes all the complications and further treatments that are considered in the decision model, for a total of 18 health states, which are reported in Table 1:

**Table 1:** The health states to be valued with UCs

Health state ID	State label	SNOMED code(s)*
1	TLM / TORS and lymph node resection	10724008|Microsurgery|118438002|Trans-oral approach|
2	The same as above, but for a re-intervention	64695001|Repeat elective|10724008|Microsurgery|118438002|Trans-oral approach|
3	Radiotherapy (RT) after surgery	169351001|Radiotherapy: infuse head/neck|262061000|Postoperative|
4	Chemo-radiotherapy (CRT) after surgery	169400008|Chemo-radiotherapy: IV|262061000|Postoperative|
5	Tracheotomy long duration	48387007|Tracheotomy|
6	Gastrostomy	54956002|Gastrostomy
7	Pharyngo-cutaneous fistula	232413009|Pharyngocutaneous fistula|
8	Hospital re-admission for febrile neutropenia	409089005|Febrile neutropenia|
9	Esopharyngeal stenosis	232372008|Nasopharyngeal stenosis|
10	Osteo-radio-necrosis	109333005|Osteoradionecrosis|
11	Post-operative hemorrhages	110265006|Postoperative hemorrhage|
12	Remission after TORS/TLM	277022003|Remission|10724008|Microsurgery|118438002|Trans-oral approach|
13	Remission after TORS/TLM + adjuvant	277022003|Remission|10724008|Microsurgery|118438002|Trans-oral approach|169400008|Chemo-radiotherapy: IV|
14	Local recurrence (intervention needed)	25173007|Recurrent tumor|255470001|Local|64695001|Repeat elective|
15	Local recurrence (RT or CRT needed)	25173007|Recurrent tumor|255470001|Local|169400008|Chemo-radiotherapy: IV|
16	Regional recurrence	25173007|Recurrent tumor|410674003|Regional
17	Distant recurrence	25173007|Recurrent tumor|261007001|Distant
18	18- Palliative care	103735009|Palliative care|

^*^Post-coordination[30] has been used for states that are not natively covered in SNOMED

As an example, the following textbox reports the factsheet for scenario #4 (the other ones are available in the additional material, all of them have been proposed to the volunteers in the French version):

After explaining each scenario and addressing questions of the interviewee, the following questions are asked, implementing the RS and SG methods, respectively:

1. *On a scale of 0 to 100, where 0 represents death and 100 represents perfect health, where would you place the above scenario?* (Let UCrs be the UC elicited with this first question).

2. *Now imagine that you can choose between an adjuvant CRT and a pill that, taken at home, has the same effectiveness as the adjuvant CRT, but without side effects. However, taking the pill carries a risk of sudden death (we will go shortly in the value of the risk). Would you consider taking the pill if the risk is low enough?*

According to the standard gamble procedure, if the person does not accept even a very small risk, the interview is stopped, and the UC of the described state turns out to 1. On the contrary, if the person accepts the gamble, the first risk value proposed is (1-UCrs). As described in [22], this initialization shortens the SG elicitation procedure.

The above two questions are the same for all the 18 scenarios, resulting in the RS and SG values for each health state.

### Methods of analysis

Descriptive statistics as mean, standard deviation, and quantiles (min, q1, median, q2, max) have been calculated for each set of collected UCs, grouped by health state. Furthermore, to compare the results obtained with the two elicitation methods employed, health states have been ordered in a ranked list, and correlation between SG- and RS-derived UCs has been analyzed using non-parametric statistical tests. Statistical analyses were performed to investigate correlations of elicited UCs and patient demographics and profile characteristics. All statistical analyses have been performed with R (https://www.R-project.org/).

Oropharyngeal carcinomas (OPCs) represent a major health problem. The oropharynx is the region of the throat behind the mouth. It contains, as main structures, the two palatine tonsils on each side and the base of the tongue. These areas play a major role in swallowing and breathing. Unfortunately, this region can be the site of cancer. The most common cancer found in the oropharynx is squamous cell carcinoma. … Patients with OPC associated with HPV tend to be young, non-smoking men.

The possible treatments.

The type of treatment that can be offered to patients suffering from OPC is of three types: a surgical intervention which removes the cancerous tissue, radiotherapy (RT), which induces the death of cancer cells, and finally chemotherapy (CT) in the form of a medicine (taken intravenously) that attacks cancer cells all over the body through the bloodstream. How these treatment modalities are chosen and combined depends on the stage of the cancer, the availability of treatment, and the patient’s preferences. The most common methods and combinations are:

- Surgery only.

- Radiotherapy only.

- Radiotherapy and Chemotherapy (Primary concurrent chemoradiation – CCRT).

- Surgery followed by radiotherapy.

- Surgery followed by CCRT.

When radiotherapy or chemo-radiation are added after surgery, this treatment is called “adjuvant”. In the case of adjuvant radiation therapy, the radiation dose is reduced compared to primary radiation therapy alone.

In order to improve functional recovery after surgery, i.e. your ability to eat, breathe and speak normally, new techniques have been developed. They consist of fully endoscopic approaches to the tumor by mouth, thus avoiding access through the neck and reducing the morbidity associated with access. This leads to a much faster recovery after surgery and a better functional result. The tumor is visualized with endoscopes and microscopes.

The two main endoscopic techniques practiced today are trans-oral robotic surgery (TORS) and trans-oral laser microsurgery (TLM). For TORS, a retractor is used to open the mouth to create space for the robotic camera and surgical instruments. Then, the surgeon uses a surgical robot to view and access the structures of the pharynx (back of the throat). The tumor is removed with an electric knife. In TLM, retractors are also used to open the mouth and access the throat, but with this technique, the surgeon uses a microscope and a laser to remove the tissue.

Both techniques show pros and cons. When TLM is performed, the visual field is usually quite small …this can compromise the correct assessment of surgical margins and trigger unnecessary adjuvant therapy. However, the precision of TLM is exceptionally high ….

TORS, on the contrary, allows for resection in one piece based on better visualization with the available endoscopes and cutting instruments…. Therefore, the analysis of surgical margins is more precise. On the contrary, the accuracy of the dissection, particularly at the deep margins, may not be as satisfactory as with the laser and the microscope.

Surgical approaches to the oropharynx are generally associated with dissection of the neck lymph nodes in order to remove the nodes eventually carrying tumor cells. An incision in the neck is made to allow the surgeon to remove the lymph nodes potentially contaminated with cancer cells.

Scenario # 4: Chemoradiotherapy (CRT) after surgery.

*Imagine you have carcinoma of the oropharynx and your specialist recommends that you have a CRT after surgery. For this treatment, you will be treated every day (around 45 min) during the week, except on weekends. The treatment will last an average of 6 weeks. Side effects can occur at any time during or after radiation therapy. You may develop side effects months or years after radiotherapy. Most side effects go away on their own or can be treated, but some side effects may last longer or become permanent. During treatment, you will receive, usually three times, in addition to radiotherapy, chemotherapy. Chemotherapy may create additional side effects. Temporary side effects during treatment are:*

*- Irritation of the pharynx*

*- Swallowing problems (you may need a feeding tube in 25% of cases)*

*- Dermatitis (inflammation of the skin, shown in figure a) of the neck (96%)* 

*- Nausea / vomiting (27%).*

*- Asthenia during and immediately after treatment*

*- Tingling of the arms or legs (25%)*

*- Hearing loss (25%)*

*- Fall in the number of white blood cells (50%) which may require hospitalization*

*The long-term side effects that you may experience, and*

*that are more common than with radiotherapy only, are:*

*- Permanent swallowing difficulties and pulmonary infections which may require frequent hospitalizations* 

*- Dry mouth.*

*- Persistent thickening and hardening of*

*the neck skin*

*In most cases, you can return to your*

*previous life after three months. However,*

*if swallowing difficulties persist*

*(10% of cases), you may need a*

*gastrostomy tube (shown in the figure). This device will serve the same purpose as the feeding tube in the nose, except that it will be surgically inserted directly through the skin into your stomach. It is much more comfortable to carry than the feeding tube in the nose. Insertion is a simple procedure, which can be performed under general or local anesthesia.*

## Results

As mentioned, 47 Swiss individuals, mean age 40.8 years (range 21–68 years, standard deviation 14.79), were enrolled for the study and interviewed in the second trimester of 2019. A complete interview took from 18 to 69 min (43.5 ± 12.5 min). Table 2 summarizes the characteristics of the respondents. Table 3 shows the summary statistics of the SG-elicited UCs and RS values for the different scenarios. We report mean and standard deviation to facilitate comparison with other possible values reported in the literature, and median and quartiles to reflect the non-normal distribution very skewed towards 1 for the SG method.

**Table 2:** Characteristics the study population

	N (min–max)	% (SD)
**sex**		
Male	14	30
Female	33	70
**Education**		
Secondary school	7	15
High school	4	8
University	36	77
**Family situation**		
has_dependant_child	7	15
has_adult_non_self-sufficient_dependant	2	2
none_of_the_above	38	83
**Total number of interviewees**	47	100

**Table 3:** The values elicited with standard gamble and rating scale methods

		Standard Gamble (UCs)							Rating Scale						
ID	Scenario	Mean	sd	Max	Min	median	q1	q3	Mean	sd	Max	Min	median	q1	q3
1	TLM/TORS intervention (first month)	0.902	0.203	1.000	0.245	1.000	0.968	1.000	0.546	0.200	1.000	0.100	0.510	0.395	0.725
2	Re-intervention	0.872	0.249	1.000	0.075	1.000	0.919	1.000	0.441	0.192	0.790	0.070	0.400	0.290	0.568
3	RT after intervention	0.850	0.275	1.000	0.040	1.000	0.880	1.000	0.448	0.193	0.840	0.050	0.400	0.300	0.630
4	CRT after intervention	0.794	0.317	1.000	0.030	1.000	0.548	1.000	0.334	0.172	0.650	0.030	0.300	0.200	0.500
5	Tracheotomy long duration	0.852	0.271	1.000	0.020	1.000	0.895	1.000	0.406	0.189	0.800	0.080	0.400	0.255	0.500
6	Gastrostomy	0.916	0.209	1.000	0.095	1.000	0.990	1.000	0.501	0.214	0.900	0.070	0.550	0.350	0.665
7	Pharyngo-cutaneous fistula	0.932	0.194	1.000	0.025	1.000	1.000	1.000	0.496	0.205	0.850	0.100	0.500	0.345	0.680
8	Hospital re-admission for febrile neutropenia	0.954	0.140	1.000	0.330	1.000	1.000	1.000	0.449	0.211	0.840	0.080	0.460	0.275	0.615
9	Esopharyngeal stenosis	0.826	0.284	1.000	0.000	0.975	0.833	1.000	0.322	0.180	0.790	0.000	0.300	0.185	0.420
10	Osteo-radio-necrosis	0.791	0.302	1.000	0.005	0.915	0.758	1.000	0.274	0.164	0.620	0.050	0.250	0.125	0.400
11	post-operative hemorrhages	0.910	0.203	1.000	0.120	1.000	0.970	1.000	0.542	0.204	0.950	0.120	0.500	0.395	0.680
12	remission after TORS/TLM	0.980	0.099	1.000	0.340	1.000	1.000	1.000	0.763	0.177	1.000	0.290	0.800	0.695	0.900
13	Remission after TORS/TLM + adjuvant	0.957	0.151	1.000	0.125	1.000	1.000	1.000	0.620	0.174	0.920	0.200	0.650	0.500	0.700
14	Local recurrence (intervention needed)	0.755	0.316	1.000	0.005	0.900	0.525	1.000	0.250	0.149	0.650	0.000	0.240	0.150	0.350
15	Local recurrence (RT or CRT needed)	0.771	0.302	1.000	0.005	0.935	0.598	1.000	0.264	0.134	0.620	0.000	0.250	0.160	0.355
16	Regional recurrence	0.859	0.283	1.000	0.005	1.000	0.878	1.000	0.378	0.188	0.790	0.030	0.350	0.250	0.500
17	Distant recurrence	0.307	0.350	1.000	0.000	0.100	0.015	0.513	0.108	0.109	0.500	0.000	0.100	0.040	0.130
18	Palliative care	0.213	0.336	1.000	0.000	0.010	0.005	0.320	0.072	0.086	0.400	0.000	0.050	0.015	0.090

**Fig. 1:**
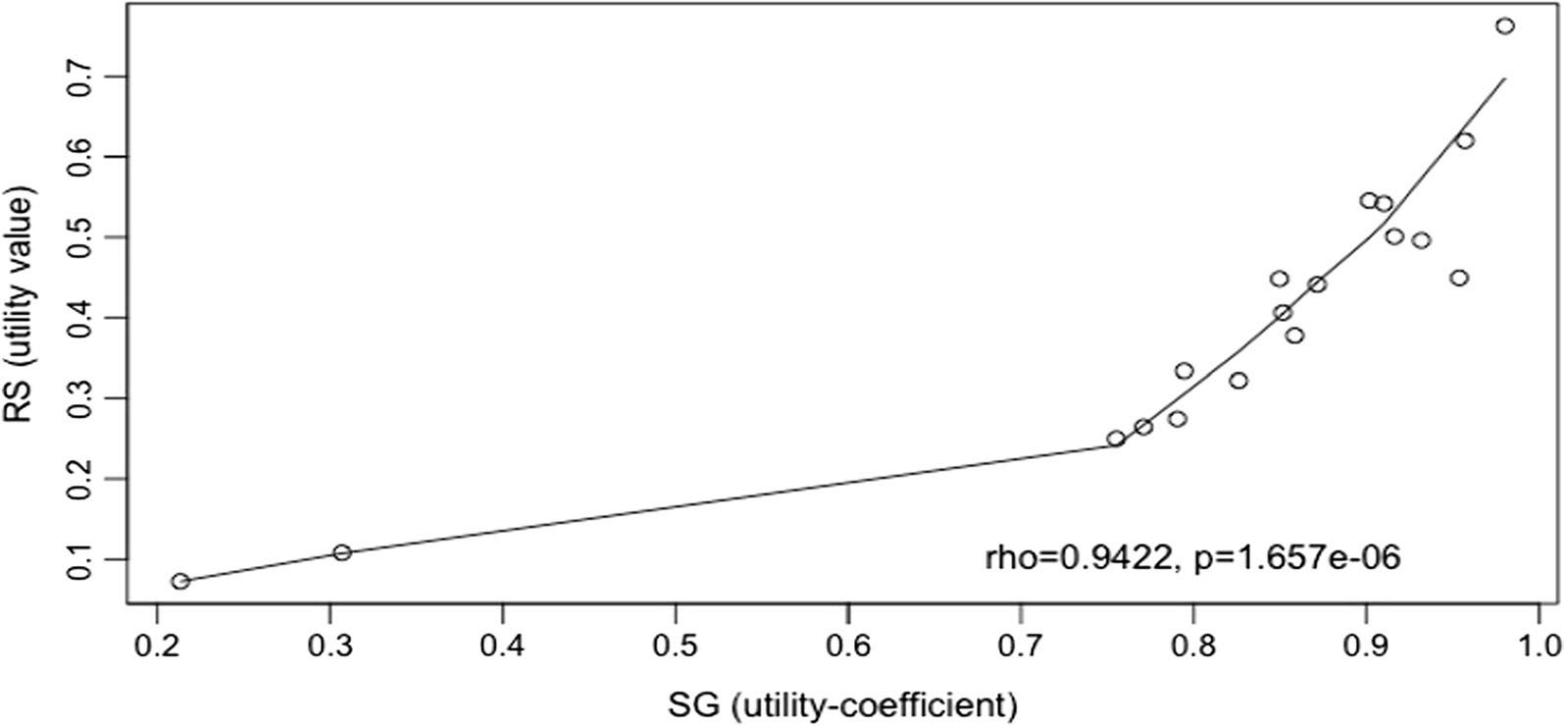
Correlation plot of mean SG utilities and RS values

**Fig. 2:**
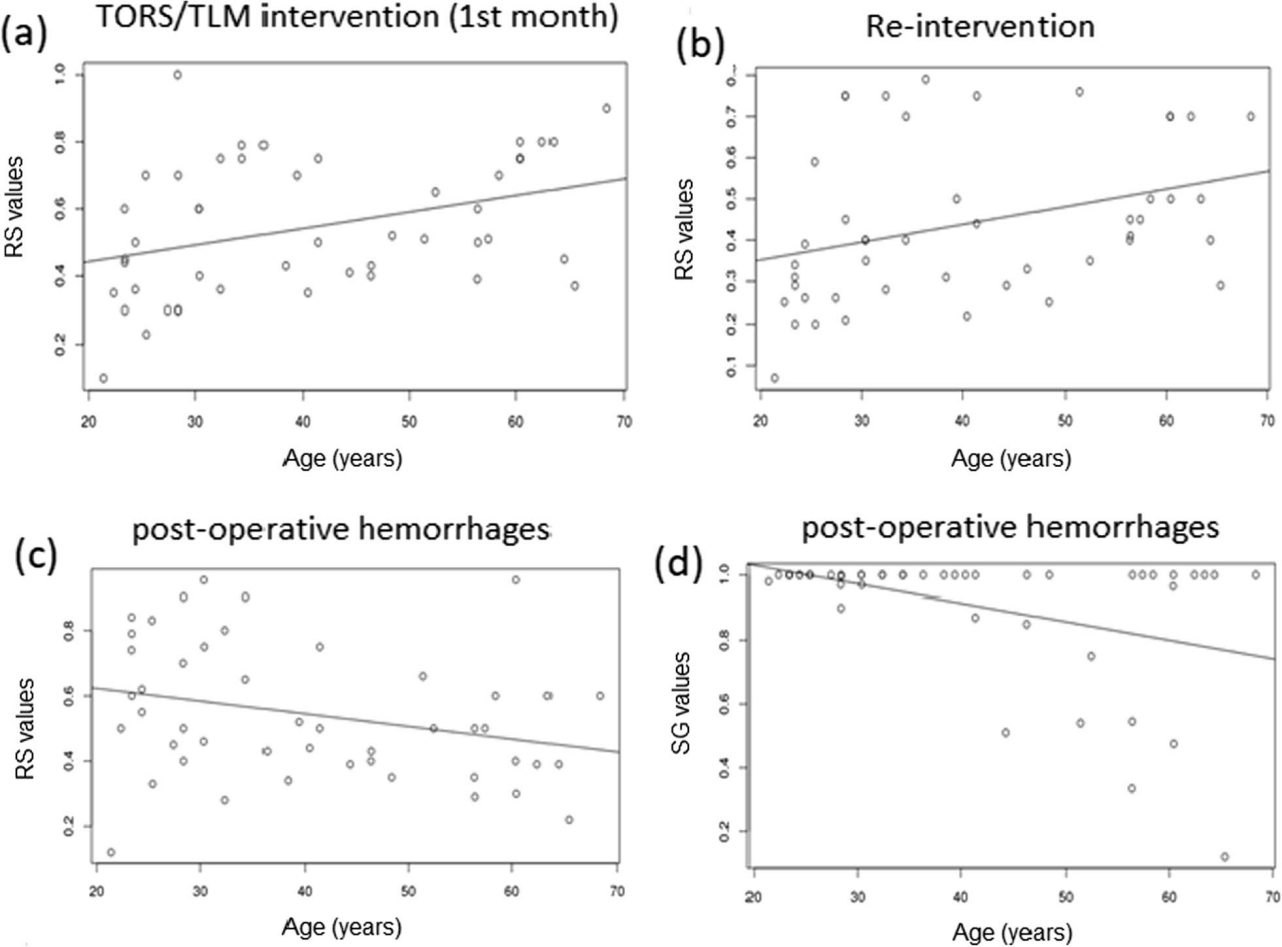
Correlation plots of RS values (**a**, **b**, **c**) and SG utilities (**d**) with age for scenarios 1 (**a**), 2 (**b**) and 11 (**c**, **d**)

Considering all the elicited values, RS and SG values showed a good correlation (Fig. 1). The smoothing line has been fitted using LOWESS [31] which is an algorithm for robust locally weighted regression (Fig. 2).

Considering the mean values, the 18 health states were ranked in the same order by the two methods 9 out of 18 times. Table 4 reports the ordering of elicited UCs from lowest (e.g. palliative care) to highest (remission).

**Table 4:** Ranking of the different health states according to values elicited with SG and RS. Grey background highlights the differences in ranking

Order #	SG ordering (low to high)	RS ordering (low to high)
1	Palliative care	Palliative care
2	Distant recurrence	Distant recurrence
3	Local recurrence (intervention needed)	Local recurrence (intervention needed)
4	local recurrence (RT or CRT needed)	local recurrence (RT or CRT needed)
5	Osteo-radio-necrosis	Osteo-radio-necrosis
6	CRT after intervention	Esopharyngeal stenosis
7	Esopharyngeal stenosis	CRT after intervention
8	RT after intervention	Regional recurrence
9	Tracheotomy long duration	Tracheotomy long duration
10	Regional recurrence	Re-intervention
11	Re-intervention	RT after intervention
12	TLM/TORS intervention (first month)	Hospital re-admission for febrile neutropenia
13	post-operative hemorrhages	Pharyngo-cutaneous fistula
14	Gastrostomy	Gastrostomy
15	Pharyngo-cutaneous fistula	post-operative hemorrhages
16	Hospital re-admission for febrile neutropenia	TLM/TORS intervention (first month)
17	Remission after TORS/TLM + adjuvant	Remission after TORS/TLM + adjuvant
18	remission after TORS/TLM	remission after TORS/TLM

In order to assess the need for tailoring decision models according to some population characteristics, we investigated the correlation of the elicited values with the respondent profile variables (gender, age, profession, marital status, education level). No significant difference was found between males and females (Wilcoxon rank sum test *p* value: 0.1824). With respect to age, no significant correlation was found with UCs elicited with SG (unless a slight negative correlation for the state 11- post-operative hemorrhages, *p* value = 0.08338, rho − 0.255219). Considering RS values, significant direct correlations were found with age for states 1 (*p *value = 0.002363, rho 0.4330367) and 2 (*p* value = 0.002985, rho 0.4282678), while significant negative correlation was found for state 11 (*p* value = 0.0456, rho − 0.2930534), giving support to the above finding with SG.

With respect to profession, unemployed people show significantly lower UCs for 4 health states (1,2,8 and 11). While people employed in commercial activities show significantly lower UCs for 3 health states (1,8 and 12). No other significant correlations were found between profession and SG utilities. Regarding marital status, the only nearly significant difference was found for state 17 with lower SG UCs elicited from married people. No differences were found when considering RS values. No significant differences were found when comparing elicited values in people with different education level.

Comparing our results with other, similar studies, Table 5 summarizes the relationship with UCs elicited in the present study and the study by de Almeida[15] on a North American population that, as mentioned in “Background” section, is the only study reporting UCs directly comparable with the ones in our study.

**Table 5:** comparison of the UCs elicited with SG method in the present study and the study from de Almeida et al. [15]. Mean and 95% CI are reported

		de Almeida et al			Present study		
IDscenario	Scenario	Mean UC	CI low	CI high	Mean UC	CI low	CI high
1	TLM/TORS intervention (first month)	0.95	0.94	0.97	0.90	0.84	0.96
2	Re-intervention	0.94	0.91	0.97	0.87	0.80	0.94
3	RT after intervention	0.89	0.85	0.93	0.85	0.77	0.93
4	CRT after intrevention	0.89	0.89	0.93	0.79	0.70	0.89
5	Tracheotomy long duration	0.85	0.80	0.91	0.85	0.77	0.93
6	Gastrostomy	0.89	0.85	0.94	0.92	0.86	0.98
7	Pharyngo-cutaneous fistula	0.89	0.85	0.94	0.93	0.88	0.99
8	Hospital re-admission for febrile neutropenia	0.96	0.94	0.98	0.95	0.91	0.99
9	Esopharyngeal stenosis	0.85	0.80	0.90	0.83	0.74	0.91
10	Osteo-radio-necrosis	0.85	0.81	0.90	0.79	0.70	0.88
11	post-operative hemorrhages	NA	NA	NA	0.91	0.85	0.97
12	remission after TORS/TLM	0.96	0.94	0.98	0.98	0.95	1.01
13	Remission after TORS/TLM + adjuvant	0.95	0.93	0.98	0.96	0.91	1.00
14	Local recurrence (intervention needed)	0.82	0.77	0.87	0.76	0.66	0.85
15	local recurrence (RT or CRT needed)	0.88	0.84	0.91	0.77	0.68	0.86
16	Regional recurrence	0.94	0.91	0.97	0.86	0.78	0.94
17	Distant recurrence	0.57	0.50	0.64	0.31	0.21	0.41
18	Palliative care	0.42	0.34	0.50	0.21	0.12	0.31

## Discussion

Our study covers UCs of all relevant complications, treatments or health state change (e.g., remission/relapse) after Head and Neck trans-oral surgey, in agreement with the literature review by Adelstein et al. [32]. Moreover, our study addresses the lack of UCs for treatment-related complications highlighted by Meregaglia et al. [14].

There is some debate as to whether patients or healthy individuals of the general population should be interviewed to derive UCs. On one hand, patients are more aware about their health state and how it affects their life, and on the other hand, the general population is probably more suitable to decide on the relative value of that health state in comparison to other comparable states as these individuals are not personally biased by their existing state. Moreover, patients tend to develop adaptive behaviors to cope with a certain heath state, thus overestimating the true utility of that state [18]. Several advisory bodies including the United States Public Health Services panel recommend using members of the general public to elicit utilities [11, 33, 34] [35]. For these reasons, we elicited UCs by interviewing healthcare professionals, on a voluntary basis. Although these individuals are not patients, they have a more nuanced understanding of the patients’ health states following treatment.

Another discussion topic is represented by the elicitation methods. We used direct methods (SG and RS). As mentioned in [16] indirect measures like EQ-5D may be more capable of discriminating different subsets of patients. However, there are limitations in using EQ-5D for our specific population of Swiss healthcare professionals. As a matter of fact, as reported on the EQ-5D website, the questionnaire is mainly for self-administration, and “proxy versions were developed for use in special cases where patients are mentally or physically not capable reporting on their health-related quality of life, for instance because of severe intellectual disability or mental health problems”. Moreover, for our specific aims, an appropriate value set of EQ-5D for Switzerland is not currently available for conversion of EQ-5D scores to UCs [36]. For these reasons, we did not administer EQ-5D in addition to SG and RS in our UC elicitation study.

As we reported in Table 4, RS and SG rank in the same order the 5 worst and the 2 best states. Thus showing higher agreement for poorest states. Also, a substantial agreement exists in ranking of the worse health states between SG and RS, suggesting a better reliability of the elicited values than what was previously reported [16].

Our study has some limitations. Confidence intervals for elicited UCs are wider than the ones reported in de Almeida [15]. This might suggest incrementing the number of interviewees, which would also allow performing sub-group analyses that may reveal more representative preferences. Finally, our interviewees had, in large part (36/47), a high level of instruction. Thus, a non-negligible selection bias has to be taken into account. Other selection biases in the present study are represented by the mean age of the interviewees (41 y.o.) and the sex (female 70%): indeed, the typical population of Head and Neck cancer patients are male and 65 y.o. [37]. Finally, our population was not profiled on tobacco use, which is a well-known behavioral risk factor for head and neck cancer [38].

## Conclusion

To the best of our knowledge this is the first study collecting UCs after head&neck trans-oral surgery in Switzerland. Moreover, our study collected, for the first time, UCs of health states associated to both TORS and TLM, and their treatment-related complications.

The elicited values will be used first of all in a decision model for the cost/utility analysis of TORS vs TLM from the perspective of the Swiss Health National System. Beyond the specific application, the elicited values represent useful data for further economic evaluations that will consider the same health states, some of which are general enough to be applied to different cancer-related scenarios.

## Supplementary Information

**Additional file 1.** Scenario descriptions for health-state utility elicitation.

## Data Availability

The computerized elicitation software used, and the analyses code are available upon request.

## Availability of data and material (data transparency)

Results data (utility coefficients) are available upon request.
