# Supplementary material for: Quality of life and health-related utility after trans-oral surgery for head and neck cancers
Source: Health Qual Life Outcomes. 2021 Nov 3;19:250. doi: 10.1186/s12955-021-01836-3 (PMC8565022; doi:10.1186/s12955-021-01836-3)
Supplement: Supplementary file 1 — Additional file 1. Scenario descriptions for health-state utility elicitation. [file 12955_2021_1836_MOESM1_ESM.docx]

Scénarios de traitement du cancer de l’oropharynx (COP)

**Contexte:**

Les carcinomes oropharyngés (COP) représentent un problème de santé important avec 400’000 nouveaux cas par an. L'oropharynx est la région de la gorge située à l'arrière de la bouche. Il contient comme structures principales ; les deux amygdales palatines de chaque côté et la base de la langue en arrière. Cette zone joue un rôle majeur dans la déglutition et la respiration. Malheureusement, cette région peut être le siège d’un cancer. Le cancer le plus fréquemment retrouvé dans l'oropharynx est le carcinome épidermoïde. Les facteurs de risque pour développer ce type de cancer sont le tabagisme et la forte consommation d'alcool. Cependant, une association avec le HPV (virus du papillome humain) a également été décrite, avec une incidence accrue d'environ 225% des COP liés au HPV au cours des 15 dernières années. Au contraire, l'incidence des COP négatifs pour le HPV, généralement associés au tabagisme et à la consommation d'alcool, a diminué de 50% [1, 2]. Les patients atteints de COP associés au HPV ont tendance à être des hommes jeunes et non-fumeurs.

Le type de traitement pouvant être proposé aux patients souffrant de COP est de trois types: une intervention chirurgicale qui enlève le tissu cancéreux, une radiothérapie (RT) qui induit la mort des cellules cancéreuses et enfin une chimiothérapie (CT) sous forme d’un médicament (administré par voie orale ou intraveineuse) qui atteinte les cellules cancéreuses partout dans l’organisme via la circulation sanguine. La manière dont ces modalités de traitement sont choisies et combinées dépend du stade du cancer, de la disponibilité du traitement et des préférences du patient.

Les modalités et les combinaisons les plus courantes sont les suivantes:

- Chirurgie seule
- Radiothérapie seule
- Chirurgie associée à la radiothérapie
- Chirurgie associée à la chimiothérapie et à la radiothérapie (chimiothérapie concurrente (CCRT))
- Chimio-radiation concurrente primaire (CCRT) seule

Lorsque la radiothérapie ou la chimio-radiation doivent être ajoutées après la chirurgie, ce traitement est appelé « adjuvant ». Dans le cas d’une radiothérapie adjuvante, la dose de rayonnement est réduite par rapport à un traitement de radiothérapie seule.

Afin d'améliorer la récupération fonctionnelle après une intervention chirurgicale, c.-à-d. votre capacité à manger, respirer et parler le plus normalement possible, de nouvelles techniques ont été développées. Ils consistent en des approches entièrement endoscopiques de la tumeur par la bouche, évitant ainsi les accès par le cou et réduisant ainsi la morbidité liée à l'accès. Cela conduit à une récupération beaucoup plus rapide après la chirurgie et à un meilleur résultat fonctionnel. La tumeur est visualisée avec des endoscopes et des microscopes.

Les deux principales techniques endoscopiques pratiquées de nos jours sont la chirurgie robotique trans-orale (TORS) et la microchirurgie au laser trans-orale (TLM). Dans le TORS, un écarteur est utilisé pour ouvrir la bouche afin de créer un espace pour la caméra robotique et les instruments chirurgicaux. Ensuite, le chirurgien utilise un robot chirurgical pour visualiser et accéder aux structures du pharynx (arrière de la gorge). La tumeur est enlevée avec un couteau électrique. Dans le TLM, les rétracteurs sont également utilisés pour ouvrir la bouche et accéder à la gorge, mais avec cette technique, le chirurgien utilise un microscope et un laser pour prélever les tissus.

Quelles sont les différences entre TLM et TORS? Lorsque TLM est effectué, le champ visuel est généralement assez petit en raison des limitations du microscope et par conséquent, la tumeur doit être enlevée en morceaux. Cela peut créer une confusion sur les marges chirurgicales et déclencher des traitements adjuvants inutiles. Cependant, la précision de TLM est exceptionnellement élevée, étant donné que la résection est effectuée à fort grossissement.

Le TORS, au contraire, permet une résection en bloc (résection en une seule pièce) basée sur une meilleure visualisation avec les endoscopes disponibles et des instruments de coupe offrant un degré de liberté de mobilité supérieur. Par conséquent, l'analyse des marges chirurgicales est plus précise. Au contraire, la précision de la dissection, particulièrement au niveau des marges profondes, risque de ne pas être aussi satisfaisante qu'avec le laser et le microscope.

Les approches chirurgicales de l'oropharynx sont généralement associées à une dissection du cou (évidement ganglionnaire) afin de retirer les ganglions lymphatiques porteurs (éventuellement) de cellules tumorales. Pour cela, une incision dans le cou doit être pratiquée pour permettre au chirurgien de retirer les ganglions lymphatiques potentiellement contaminés par des cellules cancéreuses.

**Contexte imaginaire: Imaginez être un patient qui consulte un médecin ORL (un oto-rhino-laryngologiste) suite au diagnostic d'un cancer de l'oropharynx. Votre médecin vous proposera différentes options de traitement et vous choisirez finalement l'option qui correspond le mieux à vos souhaits. Après une discussion suffisante avec lui, vous aurez suffisamment d’informations sur les risques et les avantages pour pouvoir prendre une décision.**

**Scénarios d'état de santé**

Scénario n.1 **Microchirurgie au laser trans-orale (TLM) / Chirurgie robotique trans-orale (TORS) et évidement ganglionnaire**

Imaginez qu'un cancer de l'oropharynx ait été diagnostiqué chez vous et que vous ayez été traité par microchirurgie au laser trans-orale (TLM) ou par chirurgie robotique trans-orale (TORS) et dissection du cou (incision dans le cou et retrait de tous les ganglions lymphatiques pouvant être intéressés par la maladie primaire). Vous resterez à l'hôpital en moyenne 3 à 7 jours et votre convalescence à la maison peut prendre 1 à 2 semaines supplémentaires.

Pendant une période de 3 semaines après la chirurgie, vous pouvez éprouver:

- La possibilité d'avoir une canule de trachéotomie temporaire [3] (5 patients sur 100 nécessitent une trachéostomie). Ce dispositif sera chirurgicalement mis en place pour vous permettre de respirer librement. C'est une incision à la base du cou qui permettra à un tube de passer directement dans votre trachée. Il est indiqué si vous présentez un œdème important de la gorge, secondaire à la chirurgie, pouvant entraîner des difficultés respiratoires. Les complications de la trachéostomie sont rares et sont les suivantes:
- Sténose trachéale (1,85% des cas): rétrécissement de la trachée consécutif à la mise en place du tube de trachéotomie pouvant nécessiter une intervention chirurgicale pour être corrigé.
- Saignements (0,8% des cas) ou infection (0,44% des cas) autour de la trachéotomie.
- Après retrait de la canule, persistance d'une petite communication entre la trachée et la peau, appelée fistule trachéo-cutanée dans 0,53% des cas, qui peut facilement être corrigée chirurgicalement.
- Obstruction ou déplacement de la canule pouvant causer une détresse respiratoire et nécessiter des soins médicaux en urgence dans 0,35% des cas.


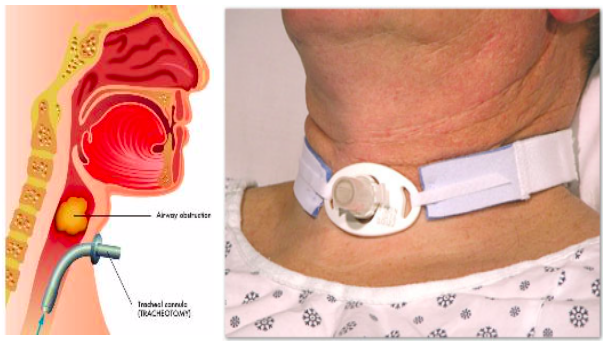


*Figure 1. Schéma d'une trachéotomie et image d'une canule en place*

- La possibilité de recourir à une sonde nasogastrique (10%): ce dispositif est mis en place par une infirmière au chevet du patient ou lors de la chirurgie pour vous permettre de vous nourrir en cas de difficulté à avaler après la chirurgie. La raison de ces difficultés peut être une douleur ou des difficultés mécaniques à avaler. Ce tube de petit calibre entrera dans votre nez et ira directement dans votre estomac. Ce n'est pas douloureux, mais cela peut être désagréable au début. Les complications possibles sont [4]:
  - Nausée: 10-20% des cas
  - Diarrhée: 30% des cas
  - Chute du tube et nécessité de le remettre en place: 25% des cas.

*Figure 2. Sonde nasogastrique et schéma d'une gastrostomie*


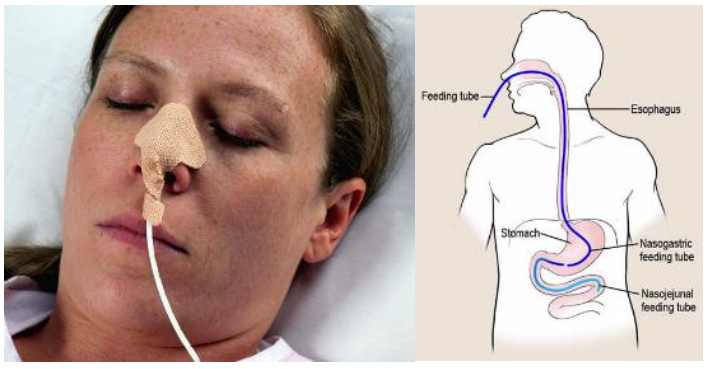


Vous pouvez également avoir d'autres effets secondaires:

- Mal de gorge et difficulté à avaler (50% des cas) [5]
- Diète liquide ou semi liquide (environ 1 semaine), composée de yaourt, potages, purée de légumes, etc.
- Faiblesse de l'épaule due à la chirurgie (20% des cas) [6]
- Difficulté à parler en raison d'une voix de mauvaise qualité (environ 1 semaine)
- Fatigue, cependant vous devriez pouvoir effectuer la plupart des activités quotidiennes à partir d'une semaine après la chirurgie

Après environ un mois, vous pourrez retrouver un état de santé normal ou nécessiter une réintervention du même type et / ou un traitement adjuvant, ce qui entraîne des effets indésirables supplémentaires, notamment une aggravation de la déglutition.

Mais concentrons-nous maintenant sur cette première intervention et ses conséquences.


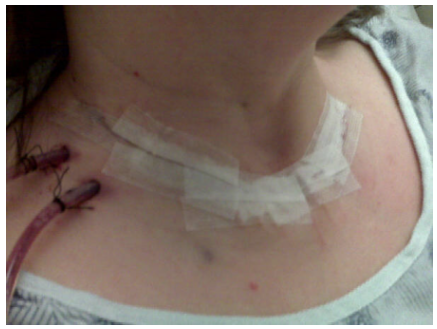


*Figure 3. État du cou le premier jour de l'opération avec un pansement couvrant l'incision et deux drains qui resteront en place pendant 2 à 4 jours*

**Questions pour l'élicitation d'utilité TLM / TORS (première intervention):**

1. Sur une échelle de 0 à 100, où 0 représente le décès et 100 la santé parfaite, où placeriez-vous le scénario ci-dessous? *Le patient choisit une valeur h et l'intervieweur calcule y = h / 100.*
2. Imaginez que vous puissiez choisir entre une chirurgie TLM / TORS et une pilule prise à la maison qui a la même efficacité que la chirurgie, mais sans effets secondaires ni complications. Cependant, prendre la pilule comporte un risque de X% de mort subite (mort immédiate). Accepteriez-vous ce risque?

*L’intervieweur commence par proposer un risque égal à 1-y, puis augmente ou diminue le risque en fonction des réponses de l’interviewé, jusqu’à ce que l’interviewé montre une certaine incertitude dans sa réponse. À ce stade, l'utilité du scénario est calculée en tant que 1-risque. Cette procédure sera la même pour tous les scénarios, elle ne sera donc pas réécrite.*

Scénario n.2 **Second TLM ou TORS (ré-intervention)**

Maintenant, imaginez que vous ayez déjà subi la première intervention et qu’après 2-3 semaines, vous deviez subir une ré-intervention avec les mêmes effets secondaires. Cette situation se produit lorsque l'analyse histologique et microscopique de la tumeur retirée lors de la première intervention chirurgicale montre que les cellules tumorales sont trop proches de l'incision pratiquée pour retirer la tumeur. Pour être sûr de retirer efficacement tout le tissu tumoral, il faut quelques millimètres entre la tumeur et cette incision. Si vous deviez choisir entre la ré-intervention ou la pilule « magique », le risque que vous avez mentionné avant (X%) (c'est-à-dire le risque de décès avec la pilule) changerait-il dans cette situation?

Scénario n.3 **Radiothérapie (RT) après chirurgie**

Imaginez que vous avez un cancer de l’oropharynx et que votre spécialiste vous recommande de subir une radiothérapie après la chirurgie. Pour ce traitement, vous serez traité tous les jours (environ 45 minutes) pendant la semaine, sauf le week-end. Le traitement durera en moyenne 6 semaines. Les effets secondaires [7] peuvent survenir à tout moment pendant ou après la radiothérapie. Il est possible que vous développiez des effets secondaires des mois ou des années après la radiothérapie. La plupart des effets secondaires disparaissent d'eux-mêmes ou peuvent être traités, mais certains effets secondaires peuvent durer plus longtemps ou devenir permanents.

Les effets secondaires temporaires au cours du traitement sont les suivants:

- Irritation du pharynx
- Problèmes de déglutition (vous aurez peut-être besoin d'une sonde d'alimentation dans 25% des cas)
- Dermatite (inflammation de la peau) du cou
- Nausées, vomissements
- Asthénie, pendant et immédiatement après le traitement

Les effets secondaires à long terme que vous pouvez ressentir sont les suivants:

- Difficultés à la déglutition permanentes et infections pulmonaires pouvant nécessiter des hospitalisations fréquentes
- Bouche sèche
- Epaississement et indurations persistants de la peau du cou

Dans la plupart des cas, vous pouvez revenir à votre vie antérieure après trois mois. Cependant, si les difficultés de déglutition persistent, vous aurez peut-être besoin d'une sonde de gastrostomie. Ce dispositif remplira le même objectif que le tube d’alimentation dans le nez, sauf qu’il sera inséré chirurgicalement directement à travers la peau dans votre estomac. Il est beaucoup plus confortable à porter que le tube d'alimentation dans le nez. L'insertion est une procédure simple, qui peut être réalisée sous anesthésie générale ou locale. Le risque de recourir à une gastrostomie est faible, inférieur à 0,05% [8].


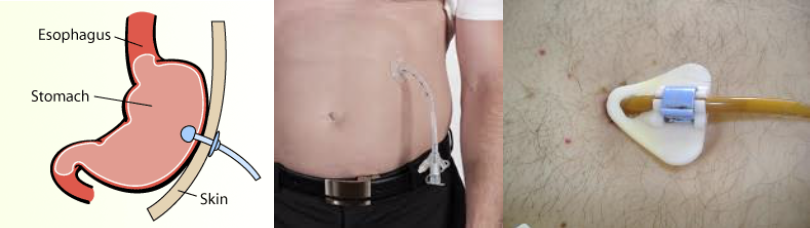


*Figure 4. Placement, schéma et image en gros plan d'un tube de gastrostomie*


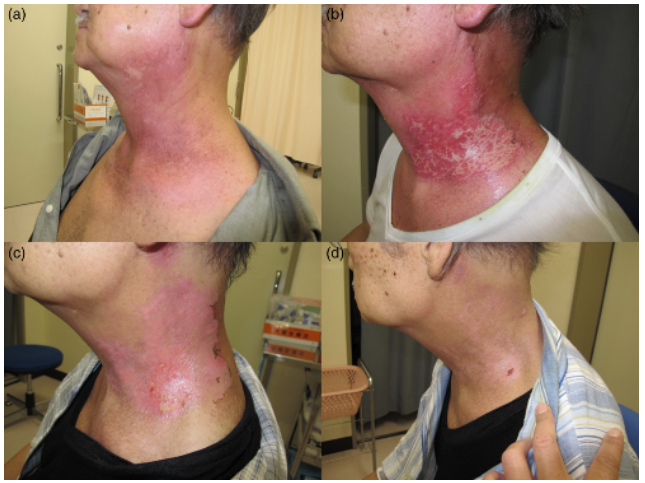


*Figure 5. Etapes typique de changement dans le temps d'une dermatite secondaire à la radiothérapie*

**Questions:**

1. Sur une échelle de 0 à 100, où 0 représente le décès et 100 la santé parfaite, où placeriez-vous le scénario ci-dessus?
2. Imaginez maintenant que vous pouvez choisir entre une RT adjuvante et une pilule qui, prise à la maison, a la même efficacité que la RT adjuvante, mais aucun effet secondaire. Cependant, prendre la pilule comporte un risque de X% de mort subite. Préférez-vous prendre la pilule et accepter ce risque?

Scénario n.4: **Chimio-radiothérapie (CRT) après une chirurgie**

Imaginez que vous avez un carcinome de l’oropharynx et que votre spécialiste vous recommande de subir un CRT après la chirurgie. Pour ce traitement, vous serez traité tous les jours (environ 45 minutes) pendant la semaine, sauf le week-end. Le traitement durera en moyenne 6 semaines. Les effets secondaires [9] peuvent survenir à tout moment pendant ou après la radiothérapie. Il est possible que vous développiez des effets secondaires des mois ou des années après la radiothérapie. La plupart des effets secondaires disparaissent d'eux-mêmes ou peuvent être traités, mais certains effets secondaires peuvent durer plus longtemps ou devenir permanents. Au cours du traitement, vous recevrez, généralement trois fois, en plus de la radiothérapie, une chimiothérapie. La chimiothérapie pour créer des effets secondaires supplémentaires.

Les effets secondaires temporaires au cours du traitement sont les suivants [9]:

- Irritation du pharynx
- Problèmes de déglutition (vous aurez peut-être besoin d'une sonde d'alimentation dans 25% des cas)
- Dermatite (inflammation de la peau) du cou (96%)
- Nausées / vomissements (27%)
- Asthénie pendant et immédiatement après le traitement
- Fourmillements des bras ou des jambes (25%)
- Perte auditive (25%)
- Chute du nombre de globules blancs (50%) pouvant nécessiter une hospitalisation

Les effets secondaires à long terme que vous pourriez ressentir sont:

- Difficultés de déglutition permanentes et infections pulmonaires pouvant nécessiter des hospitalisations fréquentes (ceci est plus fréquent qu'avec la radiothérapie uniquement)
- Bouche sèche (ceci est plus fréquent qu'avec RT seulement)
- Epaississement persistant et induration de la peau du cou (ceci est plus fréquent que pour RT seulement)

Dans la plupart des cas, vous pouvez revenir à votre vie antérieure après trois mois. Cependant, si les difficultés de déglutition persistent, vous aurez peut-être besoin d'une sonde de gastrostomie. Ce dispositif remplira le même objectif que le tube d’alimentation dans le nez, sauf qu’il sera inséré chirurgicalement directement à travers la peau dans votre estomac. Il est beaucoup plus confortable à porter que le tube d'alimentation dans le nez. L'insertion est une procédure simple, qui peut être réalisée sous anesthésie générale ou locale. Le risque de recourir à une gastrostomie peut atteindre 10% [8].


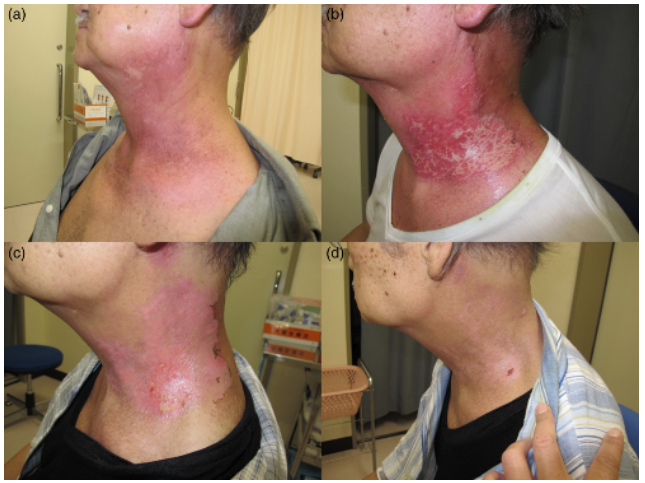


*Figure 7. Etapes typique de changement dans le temps d'une dermatite secondaire à la radiothérapie*


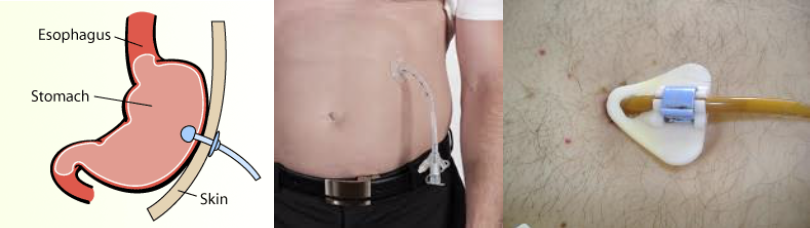


*Figure 6. Placement, schéma et image en gros plan d'un tube de gastrostomie*

**Questions:**

1. Sur une échelle de 0 à 100, où 0 représente le décès et 100 la santé parfaite, où placeriez-vous le scénario ci-dessus?
2. Imaginez maintenant que vous pouvez choisir entre un CRT adjuvant et une pilule qui, prise à la maison, a la même efficacité que le CRT adjuvant, mais sans effets secondaires. Cependant, prendre la pilule comporte un risque de X% de mort subite. Préférez-vous prendre la pilule et accepter ce risque?

Scénario n.5 : **Trachéotomie de longue durée**

Après le traitement, vous avez besoin d’un tube de trachéotomie permanent. Ce dispositif sera chirurgicalement mis en place pour vous permettre de respirer librement. C'est une incision à la base du cou qui permettra à un tube de passer directement dans votre trachée. Il est indiqué en cas de difficultés respiratoires secondaires à la chirurgie ou à la RT / CRT. Avec ce dispositif, vous pouvez toujours parler. Cependant, ce système nécessite l'aspiration de sécrétions dans la canule plusieurs fois par jour et un nettoyage de routine quotidien. Vous pouvez toujours maintenir votre régime alimentaire et manger des choses par la bouche, mais vous pouvez avoir besoin d'un régime alimentaire modifié tel que des liquides épaissis ou des aliments mous. Vous pourriez avoir des inquiétudes concernant l'apparence esthétique d'une trachéotomie. Vous êtes par ailleurs en mesure de mener à bien vos activités quotidiennes avec quelques inconvénients mineurs liés à l'entretien de ce tube. La durée d'une trachéotomie à long terme est d'au moins 1 an.


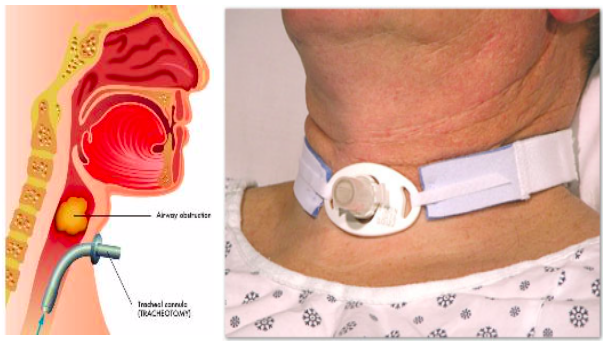


*Figure 8. Schéma d'une trachéotomie et image d'une canule en place*

**Questions**:

1. Sur une échelle de 0 à 100, où 0 représente le décès et 100 la santé parfaite, où placeriez-vous le scénario ci-dessus?
2. Imaginez maintenant que vous pouvez choisir entre une trachéotomie à long terme ou une prévention avec une pilule. Cependant, prendre la pilule comporte un risque de X% de mort subite. Préférez-vous prendre la pilule et accepter ce risque?

Scénario n.6 : **Gastrostomie**

Après le traitement de votre cancer de l'oropharynx, vous avez besoin d'une sonde de gastrostomie car vous ne pouvez pas suivre un régime complet par la bouche. Ce dispositif remplit le même objectif que le tube d’alimentation dans le nez, sauf qu’il est inséré chirurgicalement directement à travers la peau dans votre estomac. Il est beaucoup plus confortable à porter que le tube d'alimentation dans le nez. L'insertion est une procédure simple, qui peut être réalisée sous anesthésie générale ou locale. Vous devez vous administrer une alimentation adaptée par le tube plusieurs fois par jour. Le tube d'alimentation et le site nécessitent un nettoyage régulier. Vous êtes par ailleurs en mesure de mener à bien vos activités quotidiennes avec quelques inconvénients mineurs liés à l'entretien du tube.


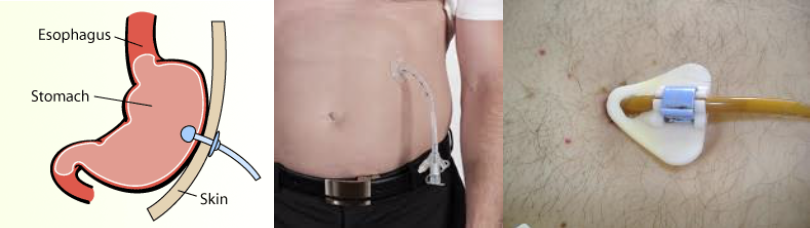


*Figure 9. Placement, schéma et image en gros plan d'un tube de gastrostomie*

**Questions**:

1. Sur une échelle de 0 à 100, où 0 représente le décès et 100 la santé parfaite, où placeriez-vous le scénario ci-dessus?
2. Imaginez maintenant que vous pouvez choisir de subir une gastrostomie prolongée ou de la prévenir avec une pilule. Cependant, prendre la pilule comporte un risque de X% de mort subite. Préférez-vous prendre la pilule et accepter ce risque?

Scénario n.7: **Fistule pharyngo-cutanée**

Après l'opération, vous présentez une fistule pharyngo-cutanée. C'est une ouverture à la peau du cou, qui est reliée à la gorge. La salive et la nourriture peuvent sortir de cette ouverture. C'est la conséquence d'une mauvaise cicatrisation de vos plaies. Pour traiter cette maladie, vous devez être hospitalisé et traité avec des antibiotiques et des changements de pansement. Fréquemment, vous aurez besoin de chirurgies supplémentaires pour fermer l'ouverture. La fistule guérira généralement dans les 3 à 6 semaines.

*Figure 10. Image d'une fistule pharyngo-cutanée*


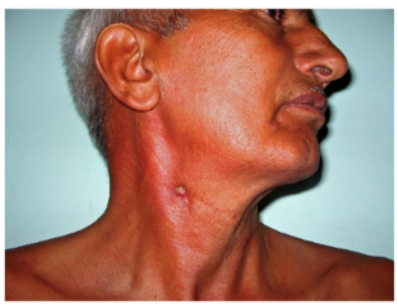


**Questions**:

1. Sur une échelle de 0 à 100, où 0 représente le décès et 100 la santé parfaite, où placeriez-vous le scénario ci-dessus?
2. Imaginez maintenant que vous pouvez choisir entre une telle fistule ou la prévenir avec une pilule. Cependant, prendre la pilule comporte un risque de X% de mort subite. Préférez-vous prendre la pilule et accepter ce risque?

Scénario n.8 : **Réadmission à l'hôpital pour neutropénie fébrile**

Pendant votre traitement du cancer de l'oropharynx par chimiothérapie, vous développez de la fièvre et vous devez être hospitalisé. Vous présentez des symptômes pseudo-grippaux au cours de cette période et une neutropénie fébrile est diagnostiquée, ce qui signifie que le niveau de vos globules blancs dans le sang est très bas dans les suites de la chimiothérapie. Cela vous rend plus vulnérable aux infections, qui peuvent être plus graves. Vous avez besoin d'antibiotiques par voie intraveineuse et de médicaments pour renforcer votre système immunitaire. Ces symptômes disparaîtront probablement en 1 à 2 semaines.

**Questions**:

1. Sur une échelle de 0 à 100, où 0 représente le décès et 100 la santé parfaite, où placeriez-vous le scénario ci-dessus?
2. Imaginez maintenant que vous pouvez choisir entre développer cette maladie ou la prévenir avec une pilule. Cependant, prendre la pilule comporte un risque de X% de mort subite. Préférez-vous prendre la pilule et accepter ce risque?

Scénario n.9: **Sténose œsophagienne**

Après une radiothérapie ou une chimio-radiothérapie adjuvante, vous développez des difficultés à avaler. Vous serez incapable de manger certains aliments solides et vous ne pourrez boire que des liquides ou manger des aliments de consistance plus molle. Ces symptômes sont liés à une sténose œsophagienne. Cette affection se caractérise par une réduction de calibre de votre œsophage, qui relie votre gorge à votre estomac. Cela peut s’aggraver progressivement au point de nécessiter une opération pour élargir le passage des aliments, après quoi vous pourrez à nouveau manger. Cette opération a un taux de complications élevé et nécessite une hospitalisation d'au moins une semaine. Même après le traitement, vous risquez toujours d'avoir des difficultés à manger des aliments plus solides. Une sténose œsophagienne restera toute votre vie, mais avec des degrés de gravité variables.


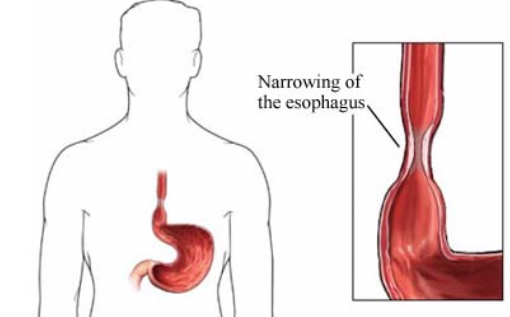


*Figure 11. Sténose œsophagienne*

**Questions:**

1. Sur une échelle de 0 à 100, où 0 représente le décès et 100 la santé parfaite, où placeriez-vous le scénario ci-dessus?
2. Imaginez maintenant que vous pouvez choisir entre une telle sténose œsophagienne ou la prévenir avec une pilule. Cependant, prendre la pilule comporte un risque de X% de mort subite. Préférez-vous prendre la pilule et accepter ce risque?

Scénario n.10: **Ostéoradionécrose**

Après une radiothérapie ou une chimio-radiothérapie adjuvante, vous développez une affection au niveau de la cavité buccale appelée ostéoradionécrose (ORN). L'ostéoradionécrose correspond à la mise à nu de l’os de la mandibule secondaire à la mort des cellules de l’os sous l'effet des radiations. L'os meurt parce que la radiation endommage ses vaisseaux sanguins. L'ostéoradionécrose est un effet secondaire rare qui se développe peu après la radiothérapie. Il se produit généralement dans la mâchoire inférieure.

Si vous souffrez d'ostéoradionécrose, vous pouvez ressentir des douleurs, un gonflement, ou une difficulté à ouvrir la mâchoire. Vous aurez un régime alimentaire modifié dans lequel vous devrez manger principalement des liquides et des aliments mous. Vous pourrez également prendre des médicaments contre la douleur et des bains de bouche pour garder la région propre. Cette condition peut durer des semaines à des mois et peut entraîner des fractures de la mâchoire. Vous pouvez également avoir besoin d'une opération majeure pour enlever l'os mort et le remplacer. Cela nécessiterait une hospitalisation de 2 à 6 semaines. L'ostéoradionécrose peut persister de 1 à 6 mois.

*Figure 12. Exemple de lésion ORN dans la bouche du patient après radiothérapie*


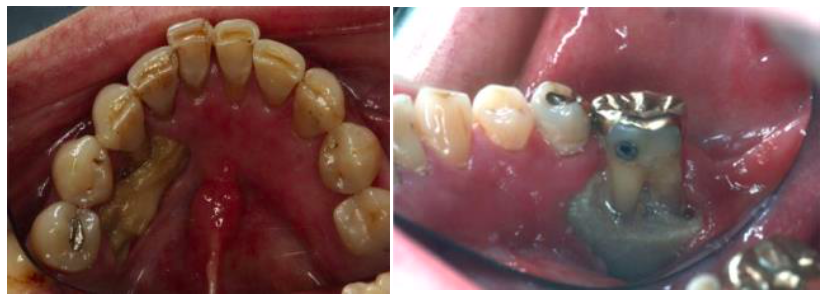


**Questions:**

1. Sur une échelle de 0 à 100, où 0 représente le décès et 100 la santé parfaite, où placeriez-vous le scénario ci-dessus?
2. Imaginez maintenant que vous pouvez choisir entre avoir l'ostéoradionécrose ou le prévenir avec une pilule. Cependant, prendre la pilule comporte un risque de X% de mort subite. Préférez-vous prendre la pilule et accepter ce risque?

Scénario n.11: **Hémorragie postopératoire [10]**

Après votre intervention au niveau de la gorge, vous êtes à risque de saignement durant les 10 premiers jours. Le plus souvent, les saignements se produisent dans les 24 heures suivant l'intervention (90% des cas), c'est pourquoi vous serez surveillé de près pendant les premiers jours après la chirurgie. Le degré de saignement varie, dans certains cas, une simple surveillance suffit et le saignement cesse tout seul. Dans d'autres situations, si le saignement est plus important, vous devrez retourner au bloc opératoire pour arrêter le saignement.

**Questions**:

1. Sur une échelle de 0 à 100, où 0 représente le décès et 100 la santé parfaite, où placeriez-vous le scénario ci-dessus?
2. Imaginez maintenant que vous pouvez choisir entre une hémorragie postopératoire ou la prévenir avec une pilule. Cependant, prendre la pilule comporte un risque de X% de mort subite. Préférez-vous prendre la pilule et accepter ce risque?

Scénario n.12 : **Rémission après TORS / TLM**

Vous avez été traité pour votre cancer de l'oropharynx avec une technique peu invasive, sans traitement adjuvant supplémentaire. On vous dit que le cancer a peu de chances de réapparaître. Vous devez donc consulter un médecin tous les trois mois pour un examen, afin d'identifier une éventuelle récidive. Vous êtes capable de mener à bien toutes vos activités. Vous n'avez aucune limite en termes de parole et de respiration. Vous pouvez rencontrer de légères difficultés à avaler quelques semaines après votre traitement. Vous pourriez être occasionnellement inquiet ou préoccupé quant à la possibilité d’une récidive du cancer.

**Questions**:

1. Sur une échelle de 0 à 100, où 0 représente le décès et 100 la santé parfaite, où placeriez-vous le scénario ci-dessus?
2. Imaginez maintenant que vous pouvez choisir de vivre dans cet état de rémission ou de prendre une pilule qui ne nécessite pas de bilan médical périodique et qui atténue également l’anxiété liée au risque de récidive. Cependant, prendre la pilule comporte un risque de X% de mort subite. Préférez-vous prendre la pilule et accepter ce risque?

Scénario n.13: **Rémission après traitement TORS / TLM + adjuvant**

Vous avez été traité pour votre cancer de l'oropharynx avec une technique peu invasive suivie d'un traitement adjuvant. Il existe un risque faible que le cancer réapparaisse, vous devez donc consulter un médecin tous les trois mois pour un examen, afin d'identifier une éventuelle récidive. Vous êtes capable de mener toutes vos activités. Vous pourriez avoir une sécheresse buccale persistante, un remaniement et un épaississement de la peau de votre cou et des difficultés à avaler. Vous pourriez être occasionnellement inquiet ou préoccupé quant à la possibilité d’une récidive du cancer.

**Questions**:

1. Sur une échelle de 0 à 100, où 0 représente le décès et 100 la santé parfaite, où placeriez-vous le scénario ci-dessus?
2. Imaginez maintenant que vous pouvez choisir de vivre dans l’état de rémission décrit ci-dessus ou de prendre une pilule qui ne vous oblige pas à passer un examen médical périodique et à vous soulager de l’anxiété liée au risque de récurrence. Cependant, prendre la pilule comporte un risque de X% de mort subite. Préférez-vous prendre la pilule et accepter ce risque?

Scénario n.14 **Récidive locale (nécessitant une intervention chirurgicale)**

Une fois le traitement de votre cancer de l'oropharynx terminé vous vous trouvez dans une phase de rémission, c.-à-d. sans cancer. Malheureusement, votre médecin vous informe que votre cancer est revenu dans votre gorge. Cette récurrence nécessite une opération majeure. Au cours de cette opération, une partie de votre pharynx sera retirée et le défaut laissé par cette opération sera reconstruit avec ce que nous appelons un lambeau. Ce lambeau est constitué de muscle et / ou de peau provenant d'un autre site de votre corps. Vous serez hospitalisé environ 15 à 20 jours après votre opération. Vous avez besoin d'une trachéotomie et d'une sonde d'alimentation pendant quelques semaines après l'opération. Parce que vous avez déjà suivi un traitement, vous présentez un risque plus élevé de complications pouvant prolonger votre séjour à l'hôpital. Pendant votre convalescence, vous ne pourrez boire ou manger par la bouche pendant au moins une à deux semaines. De plus, vous pouvez avoir des difficultés avec votre élocution, une fatigue marquée et vous pouvez vous sentir frustré de devoir faire face au retour du cancer.

**Questions**:

1. Sur une échelle de 0 à 100, où 0 représente le décès et 100 la santé parfaite, où placeriez-vous le scénario ci-dessus?
2. Imaginez maintenant que vous pouvez choisir entre subir une telle intervention chirurgicale pour une récidive locale ou prévenir tout cela avec une pilule. Cependant, prendre la pilule comporte un risque de X% de mort subite. Préférez-vous prendre la pilule et accepter ce risque?

Scénario n.15: **Récurrence locale (nécessitant une radiothérapie +/- une chimiothérapie)**

Une fois le traitement de votre cancer de l'oropharynx terminé vous vous trouvez dans une phase de rémission, c.-à-d. sans cancer. Malheureusement, votre médecin vous informe que votre cancer est revenu dans votre gorge. Cette récurrence nécessite une radiothérapie possiblement associée à une chimiothérapie. Vous avez besoin de vous rendre quotidiennement à l’hôpital (du lundi au vendredi) pour la radiothérapie. En cas d’association à une chimiothérapie, vous recevrez chaque lundi une dose de chimiothérapie.

Les effets secondaires temporaires au cours du traitement sont les suivants [9]:

- Irritation de la gorge avec des douleurs à la déglutition
- Difficulté lors de la déglutition (vous aurez peut-être besoin d'une sonde d'alimentation dans 25% des cas)
- Dermatite (inflammation de la peau) du cou (96%)
- Nausées / vomissements (27%)
- Fatigue pendant et immédiatement après le traitement
- Engourdissement des bras ou des jambes (25%)
- Perte auditive (25%)
- Diminution du nombre de globules blancs (50%) pouvant nécessiter une hospitalisation

Les effets secondaires à long terme que vous pourriez ressentir sont les suivants:

- Difficultés permanentes lors de la déglutition pouvant induire des épisodes d’infections pulmonaires pouvant nécessiter des hospitalisations fréquentes (ceci est plus fréquent qu'avec la radiothérapie uniquement)
- Bouche sèche (ceci est plus fréquent qu'avec RT seulement)
- Epaississement persistant et induration de la peau du cou (ceci est plus fréquent que pour RT seulement)

Vous pouvez ressentir une certaine frustration face au retour du cancer.

Dans la plupart des cas, vous pouvez revenir à votre vie antérieure après trois mois. Cependant, si les difficultés de déglutition persistent, vous aurez peut-être besoin d'une sonde de gastrostomie. Ce dispositif remplira le même objectif que le tube d’alimentation dans le nez, sauf qu’il sera inséré chirurgicalement directement à travers la peau dans votre estomac. Il est beaucoup plus confortable à porter que le tube d'alimentation dans le nez. L'insertion est une procédure simple, qui peut être réalisée sous anesthésie générale ou locale. Le risque de recourir à une gastrostomie peut atteindre 10% [9].


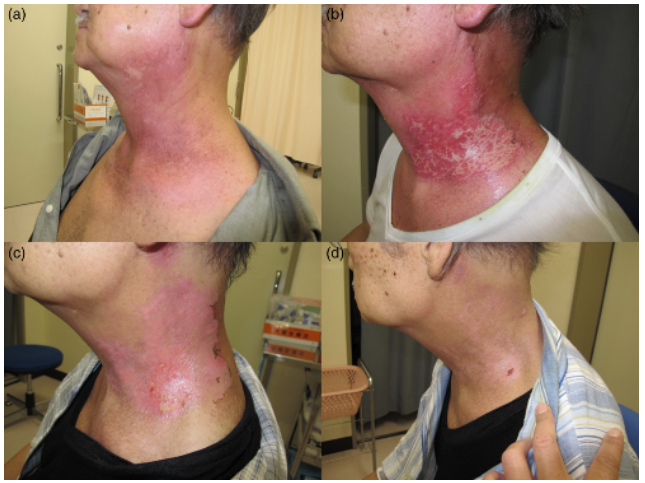


*Figure 14. Etapes typique de changement dans le temps d'une dermatite secondaire à la radiothérapie*


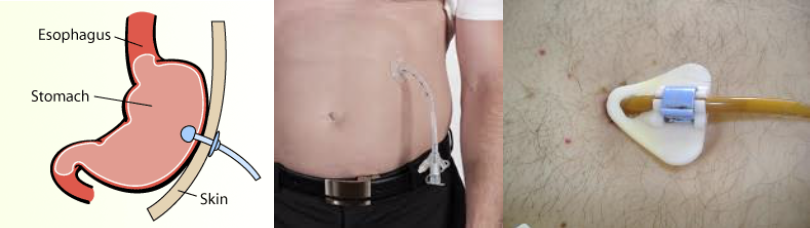


*Figure 13. Placement, schéma et image en gros plan d'un tube de gastrostomie*

**Questions**:

1. Sur une échelle de 0 à 100, où 0 représente le décès et 100 la santé parfaite, où placeriez-vous le scénario ci-dessus?
2. Imaginez que vous puissiez choisir de subir une TRC pour une récidive locale ou d’empêcher tout cela avec une pilule. Cependant, prendre la pilule comporte un risque de X% de mort subite. Préférez-vous prendre la pilule et accepter ce risque?

Scénario n.16**: Récidive régionale (nécessitant un évidement ganglionnaire)**

Une fois le traitement de votre cancer de l'oropharynx terminé vous vous trouvez dans une phase de rémission, c.-à-d. sans cancer. Malheureusement, votre médecin vous informe que votre cancer est revenu dans les ganglions lymphatiques du cou.

Cette récurrence du cancer nécessite une opération pour enlever les ganglions lymphatiques du cou appelée évidement ganglionnaire. Il s’agit d’une incision dans le cou qui permettra au chirurgien d’enlever les ganglions lymphatiques susceptibles d’être contaminés par le cancer de la gorge. Vous serez admis à l'hôpital pendant 5 à 7 jours. Vous pourriez ressentir une sensibilité et des engourdissements dans votre cou pendant environ une semaine. Vous pourriez également présenter une faiblesse au niveau de votre épaule (20% des cas). L'engourdissement peut rester plus longtemps. Après cela, vous retournerez à votre style de vie normal.


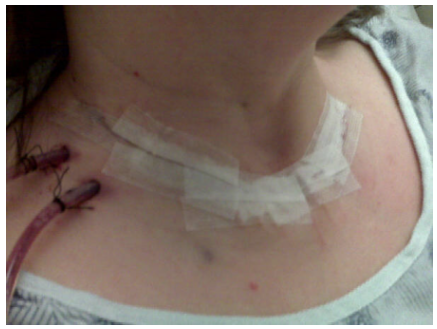


*Figure 15. État du cou le premier jour de l'opération avec un pansement couvrant l'incision et deux drains qui resteront en place pendant 2 à 4 jours*

**Questions**:

1. Sur une échelle de 0 à 100, où 0 représente le décès et 100 la santé parfaite, où placeriez-vous le scénario ci-dessus?
2. Imaginez que vous puissiez choisir de subir une telle dissection du cou pour une récidive régionale ou de l’empêcher avec une pilule. Cependant, prendre la pilule comporte un risque de X% de mort subite. Préférez-vous prendre la pilule et accepter ce risque?

Scénario n.17: **Récurrence à distance**

Une fois le traitement de votre cancer de l'oropharynx terminé vous vous trouvez dans une phase de rémission, c.-à-d. sans cancer. Malheureusement, votre médecin vous informe que votre cancer s'est propagé à un autre endroit du corps, à l'écart du cancer d'origine, on parle alors de métastases à distance. Elles peuvent se trouver dans vos poumons, votre foie ou vos os. À ce stade, la maladie est incurable et, en moyenne, l’espérance de vie est inférieure à un an. Vous aurez des symptômes tels que fatigue, douleur, dépression et perte d’appétit. Au cours des prochaines semaines ou des prochains mois, vous aurez moins d’énergie et aurez besoin d’aide pour vos activités quotidiennes.

**Questions**:

1. Sur une échelle de 0 à 100, où 0 représente le décès et 100 la santé parfaite, où placeriez-vous le scénario ci-dessus?
2. Imaginez maintenant que vous pouvez choisir de développer de telles métastases à distance ou de les prévenir avec une pilule. Cependant, prendre la pilule comporte un risque de X% de mort subite. Préférez-vous prendre la pilule et accepter ce risque?

Scénario n.18: **Soins palliatifs**

Après le traitement du cancer de l'oropharynx, vous développez une récidive agressive de votre cancer de la gorge, qui ne peut plus être guéri par une intervention chirurgicale ou une radiothérapie. Vous aurez des symptômes tels que difficulté à avaler, des maux de gorge et des saignements au niveau de la tumeur dans la bouche. Cette récidive est douloureuse. Vous serez également fatigué et déprimé. Vous aurez probablement besoin d'une trachéostomie et d'une sonde d'alimentation. Le traitement par chimiothérapie peut aider à soulager les symptômes et à prolonger légèrement l'espérance de vie à environ 8 à 10 mois. Cependant, la chimiothérapie sera associée à certains effets secondaires tels que nausées, vomissements et toxicité cutanée. Au cours des prochaines semaines ou des prochains mois, vous aurez moins d’énergie et aurez besoin d’aide pour vos activités quotidiennes.

**Questions**:

1. Sur une échelle de 0 à 100, où 0 représente le décès et 100 la santé parfaite, où placeriez-vous le scénario ci-dessus?
2. Imaginez maintenant que vous pouvez choisir entre recevoir de tels soins palliatifs ou éviter tout cela avec une pilule. Cependant, prendre la pilule comporte un risque de X% de mort subite. Préférez-vous prendre la pilule et accepter ce risque?

**Références**

[1] Tribius S, Hoffmann M. Human Papilloma Virus Infection in Head and Neck Cancer. *Dtsch Ärztebl Int* 2013; 110: 184–190.

[2] Kobayashi K, Hisamatsu K, Suzui N, et al. A Review of HPV-Related Head and Neck Cancer. *J Clin Med* 2018; 7: 241.

[3] Goldenberg D, Golz A, Netzer A, et al. Tracheotomy: changing indications and a review of 1,130 cases. *J Otolaryngol* 2002; 31: 211–215.

[4] Stroud M. Guidelines for enteral feeding in adult hospital patients. *Gut* 2003; 52: 1vii – 12.

[5] Quality of life outcomes of transoral robotic surgery with or without adjuvant therapy for oropharyngeal cancer., https://www.medscape.com/medline/abstract/28771728 (accessed 18 December 2018).

[6] Rajendra Prasad B, Sharma SM, Thomas S, et al. Assessment of shoulder function after functional neck dissection and selective neck dissection (Levels I, II, III) in patients with carcinoma of tongue: a comparative study. *J Maxillofac Oral Surg* 2009; 8: 224.

[7] TOLENTINO E de S, CENTURION BS, FERREIRA LHC, et al. Oral adverse effects of head and neck radiotherapy: literature review and suggestion of a clinical oral care guideline for irradiated patients. *J Appl Oral Sci* 2011; 19: 448–454.

[8] Vermorken JB, Mesia R, Rivera F, et al. Platinum-based chemotherapy plus cetuximab in head and neck cancer. *N Engl J Med* 2008; 359: 1116–1127.

[9] Givens DJ, Karnell LH, Gupta AK, et al. Adverse Events Associated With Concurrent Chemoradiation Therapy in Patients With Head and Neck Cancer. *Arch Otolaryngol Neck Surg* 2009; 135: 1209–1217.

[10] Pita EG, Cajelli AL, Latourrette D, et al. Transoral Surgery for Laryngeal Cancer: Bleeding Complications. *J Otolaryngol-ENT Res* 2016; 4: 1–0.
